# Cavitary rheumatoid nodules: an unusual pulmonary finding

**DOI:** 10.1590/0100-3984.2017.0182

**Published:** 2019

**Authors:** Ana Clara da Costa Geraldino, Edson Marchiori

**Affiliations:** 1 Universidade Federal do Rio de Janeiro (UFRJ), Rio de Janeiro, RJ, Brazil.

Dear Editor,

A 45-year-old female sought medical treatment at another institution complaining of a 4-month history of dry cough and dyspnea. She reported progressive worsening of the respiratory symptoms, and chest X-rays showed cavitary nodular lesions, predominantly in the periphery of the lungs. She also reported having previously been diagnosed with rheumatoid arthritis, which was treated only sporadically. The patient was admitted and underwent bronchoscopy with sputum smear microscopy, culture, and direct mycological examination, all of which were negative. Therefore, she was discharged to outpatient follow-up. Despite multiple antibiotic regimens, the clinical condition worsened and empirical treatment for tuberculosis was prescribed. The patient then developed drug-induced hepatitis, again requiring hospitalization. Computed tomography of the chest showed multiple nodular lesions, several of them cavitary, in both lungs (Figure 1). Following transesophageal echocardiography, the diagnostic hypothesis of endocarditis was rejected. The patient was then referred to our hospital for a definitive diagnosis. The test for antineutrophil cytoplasmic antibodies was negative. The patient tested positive for rheumatoid factor, although the remaining laboratory tests revealed no significant alterations. Bronchoscopy with bronchoalveolar lavage was performed, and the bronchoalveolar lavage fluid tested negative for fungi by periodic acid-Schiff staining as well as for acid-fast bacilli by Ziehl-Neelsen staining; cultures were also negative. An open lung biopsy showed a cavitary subpleural nodule with extensive central necrosis and fibrosis with a hyaline aspect at the periphery, containing histiocytes and fibroblasts, consistent with a pulmonary rheumatoid nodule. The adjacent pulmonary tissue showed a moderate amount of interstitial mononuclear inflammatory infiltrate, with pneumocyte hyperplasia and mild interstitial fibrosis, consistent with nonspecific interstitial pneumonia. During hospitalization, the patient developed respiratory failure secondary to bacterial pneumonia, and she died in the intensive care unit.

**Figure 1:**
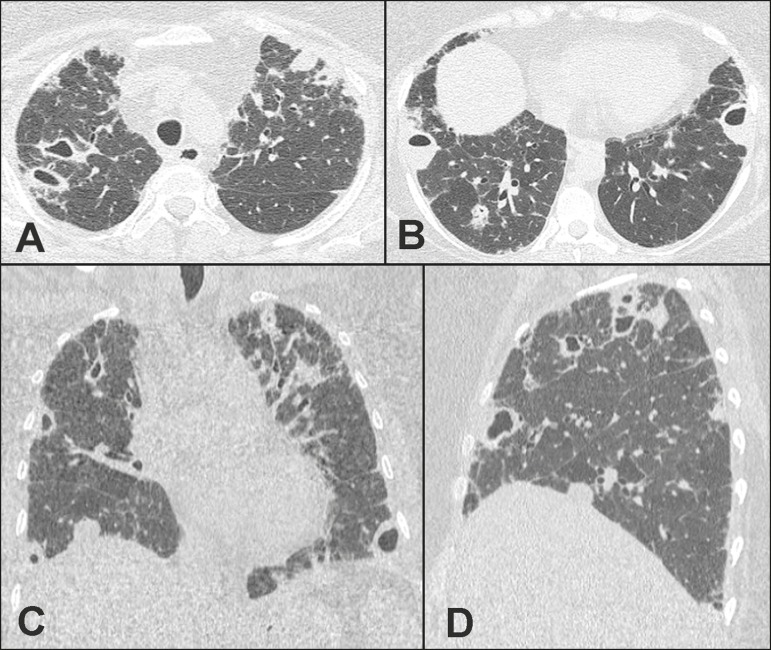
Computed tomography of the chest, in axial views (**A,B**), a coronal view (**C**), and a sagittal view (**D**), showing multiple nodular lesions with different degrees of cavitation, some with air-fluid levels. Note also the discrete subpleural opacities with reticulation.

Rheumatoid arthritis is a chronic, systemic inflammatory autoimmune disease, which is characterized by persistent inflammation of the diarthrodial joints with synovial hyperplasia that, if persistent, results in progressive joint destruction^(^^1^^,^^2^^)^. Approximately 40% of affected patients present extra-articular manifestations, pulmonary involvement being the second most common cause of death in such patients^(^^1^^-^^3^^)^.

Many recent studies published in Brazil have emphasized the importance of radiology in diagnosing thoracic diseases^(^^4^^-^^8^^)^. Rheumatoid nodules, usually subcutaneous, are the most common manifestation of rheumatoid arthritis. They are most common in male smokers and occur in approximately one third of HIV-infected patients. Although the nodules are typically found in periarticular areas exposed to pressure, they can also be found in other organs^(^^3^^,^^9^^)^. Pulmonary rheumatoid nodules are identical to the nodules found in subcutaneous tissue. They usually measure 0.5-5.0 cm in diameter, are located in peripheral areas of the upper or middle zones of the lungs, can undergo cavitation or calcification, can increase in size, and can even be spontaneously reabsorbed^(^^1^^,^^2^^)^. In most cases, they are asymptomatic and do not require specific treatment^(^^3^^,^^9^^,^^10^^)^.

Histologically, pulmonary rheumatoid nodules are similar to their extrapulmonary counterparts, with central necrosis, palisading of epithelial cells, mononuclear infiltrate, and vasculitis^(^^2^^,^^9^^)^. Pulmonary rheumatoid nodules should be differentiated from malignant and infectious processes, especially when there is only a solitary nodule. Therefore, radiological follow-up and occasionally a biopsy may be necessary to exclude malignancy^(^^2^^,^^9^^)^.

## References

[1] Yuksekkaya R, Celikyay F, Yilmaz A (2013). Pulmonary involvement in rheumatoid arthritis: multidetector
computed tomography findings. Acta Radiol.

[2] Anaya JM, Diethelm L, Ortiz LA (1995). Pulmonary involvement in rheumatoid arthritis. Semin Arthritis Rheum.

[3] Sargin G, Senturk T (2015). Multiple pulmonary rheumatoid nodules. Reumatologia.

[4] von Ranke FM, Freitas HMP, Dinoá V (2018). Congenital lobar emphysema. Radiol Bras.

[5] Oliveira DS, Araújo Filho JA, Paiva AFL (2018). Idiopathic interstitial pneumonias: review of the latest American
Thoracic Society/European Respiratory Society classification. Radiol Bras.

[6] Hochhegger B, Irion KL, Hochhegger D (2018). Pneumorrhachis as a complication of bronchial asthma: computed
tomography findings. Radiol Bras.

[7] Barbosa DL, Hochhegger B, Souza Jr AS (2017). High-resolution computed tomography findings in eight patients
with hantavirus pulmonary syndrome. Radiol Bras.

[8] Belém LC, Souza CA, Souza Jr AS (2017). Metastatic pulmonary calcification: high-resolution computed
tomography findings in 23 cases. Radiol Bras.

[9] Amital A, Shitrit D, Adir Y (2011). The lung in rheumatoid arthritis. Presse Med.

[10] Herrero HG, Sarasa MA, Marco IR (2012). Nódulos pulmonares reumatoides: forma de
presentación, métodos diagnósticos y evolución,
a propósito de 5 casos. Reumatol Clin.

